# Supplementary material for: Synthesis of Superionic Conductive Li1+x+yAlxSiyTi2−xP3−yO12 Solid Electrolytes
Source: Nanomaterials (Basel). 2022 Mar 31;12(7):1158. doi: 10.3390/nano12071158 (PMC9000703; doi:10.3390/nano12071158)
Supplement: Supplementary file 1 [file nanomaterials-12-01158-s001.zip › nanomaterials-1648639-supplementary.pdf]

nanomaterials

Supplementary Information

Synthesis of Superionic Conductive $Li_{1+x+y}Al_xSi_yTi_{2-x}P_{3-y}O_{12}$ Solid Electrolytes

Hyeonwoo Jeong [1], Dan Na [1], Jiyeon Baek [1], Sanggil Kim [1], Suresh Mamidi [1], Cheul-Ro Lee [1], Hyung-Kee Seo [2] and Inseok Seo [1,*]

1 School of Advanced Materials Engineering, Jeonbuk National University, Baekje-daero 567, Jeonju 54896, Korea; grassmarket@naver.com (H.J.); ld3310@jbnu.ac.kr (D.N.); asssa2089@naver.com (J.B.); agaleon@daum.net (S.K.); sureshmamidi@jbnu.ac.kr (S.M.); crlee7@jbnu.ac.kr (C.L.)
2 Future Energy Convergence Core Center, School of Chemical Engineering, Jeonbuk National University, Baekje-daero 567, Jeonju 54896, Korea; hkseo@jbnu.ac.kr
* Correspondence: isseo@jbnu.ac.kr; Fax: +82-63-270-2305

Table S1. Comparison of ionic conductivities and activation energies of various solid electrolytes.

Materials	σ (mS cm^{-1})	E_a (eV)	Ref
$Li_{1.4}Al_{0.4}Ti_{1.6}(PO_4)_3$ (LATP)	0.533	0.306	[1]
$Li_{1.5}Al_{0.5}Ge_{1.5}(PO_4)_3$ (LAGP)	0.521	0.34	[2]
$Li_{1.4}Al_{0.4}Ge_{0.2}Ti_{1.4}(PO_4)_3$ (LAGTP)	0.526	0.249	[3]
$Li_{1.3}Al_{0.3}Ti_{1.7}(PO_4)_{2.9}(VO_4)_{0.1}$ (LATPV)	0.26	0.30	[4]
$LiZr_2(PO_4)_3$ (LZP)	0.1	0.34	[5]
$LiTi_2(PO_4)_3$ (LTP)	0.002	-	[6]
$Li_{1.6}Al_{0.6}Sn_{1.4}P_3O_{12}$ (LASP)	0.001	-	[7]
$Li_{1.5}Al_{0.3}Si_{0.2}Ti_{1.7}P_{2.8}O_{12}$ (LASTP)	0.9455	0.226	This work

References

1. Davis III, C.; Nino, J.C. Microwave Processing for Improved Ionic Conductivity in $Li_2O–Al_2O_3–TiO_2–P_2O_5$ Glass-Ceramics. *J. Am. Ceram. Soc.* **2015**, *98*, 2422–2427, doi:https://doi.org/10.1111/jace.13638.
2. Yang, J.; Huang, Z.; Huang, B.; Zhou, J.; Xu, X. Influence of phosphorus sources on lithium ion conducting performance in the system of $Li_2O–Al_2O_3–GeO_2–P_2O_5$ glass–ceramics. *Solid State Ion.* **2015**, *270*, 61–65, doi:https://doi.org/10.1016/j.ssi.2014.12.013.
3. Zhang, P.; Wang, H.; Lee, Y.-G.; Matsui, M.; Takeda, Y.; Yamamoto, O.; Imanishi, N. Tape-Cast Water-Stable NASICON-Type High Lithium Ion Conducting Solid Electrolyte Films for Aqueous Lithium-Air Batteries. *J. Electrochem. Soc.* **2015**, *162*, A1265–A1271, doi:10.1149/2.0711507jes.
4. Chang, C.-M.; Lee, Y. Il; Hong, S.-H.; Park, H.-M. Spark Plasma Sintering of $LiTi_2(PO_4)_3$-Based Solid Electrolytes. *J. Am. Ceram. Soc.* **2005**, *88*, 1803–1807, doi:https://doi.org/10.1111/j.1551-2916.2005.00246.x.
5. El-Shinawi, H.; Regoutz, A.; Payne, D.J.; Cussen, E.J.; Corr, S.A. NASICON $LiM_2(PO_4)_3$ electrolyte (M = Zr) and electrode (M = Ti) materials for all solid-state Li-ion batteries with high total conductivity and low interfacial resistance. *J. Mater. Chem. A* **2018**, *6*, 5296–5303, doi:10.1039/C7TA08715B.
6. Aono, H.; Sugimoto, E.; Sadaoka, Y.; Imanaka, N.; Adachi, G. Ionic conductivity and sinterability of lithium titanium phosphate system. *Solid State Ion.* **1990**, *40–41*, 38–42, doi:https://doi.org/10.1016/0167-2738(90)90282-V.
7. Rusdi, H.; Mohamed, N.S.; Subban, R.H.Y. Effects of temperatures to the electrical properties of $Li_{1.6}Al_{0.6}Sn_{1.4}P_3O_{12}$ NASICON type solid electrolytes. *AIP Conf. Proc.* **2017**, *1877*, 50005, doi:10.1063/1.4999879.
